# SEC61 translocon gamma subunit is correlated with glycolytic activity, epithelial mesenchymal transition and the immune suppressive phenotype of lung adenocarcinoma: SEC61G is correlated with the glycolytic and immune phenotypes of LUAD

**DOI:** 10.3724/abbs.2024109

**Published:** 2024-07-08

**Authors:** Changshuai Zhou, Huanhuan Cui, Yuechao Yang, Lei Chen, Mingtao Feng, Yang Gao, Deheng Li, Liangdong Li, Xin Chen, Xiaoqiu Li, Yiqun Cao

**Affiliations:** 1 Department of Neurosurgery Fudan University Shanghai Cancer Center Shanghai 200032 China; 2 Department of Pathology Fudan University Shanghai Cancer Center Shanghai 200032 China; 3 Department of Oncology Shanghai Medical College Fudan University Shanghai 200032 China

**Keywords:** SEC61G, lung adenocarcinoma, tumor immune microenvironment, glycolysis, epithelial mesenchymal transition

## Abstract

Lung adenocarcinoma (LUAD) remains a predominant cause of cancer-related mortality globally, underscoring the urgency for targeted therapeutic strategies. The specific role and impact of the SEC61 translocon gamma subunit (SEC61G) in LUAD progression and metastasis remain largely unexplored. In this study, we use a multifaceted approach, combining bioinformatics analysis with experimental validation, to elucidate the pivotal role of SEC61G and its associated molecular mechanisms in LUAD. Our integrated analyses reveal a significant positive correlation between SEC61G expression and the glycolytic activity of LUAD, as evidenced by increased fluorodeoxyglucose (FDG) uptake on positron emission tomography (PET)/CT scans. Further investigations show the potential influence of SEC61G on metabolic reprogramming, which contributes to the immunosuppressive tumor microenvironment (TME). Remarkably, we identify a negative association between SEC61G expression levels and the infiltration of critical immune cell populations within the TME, along with correlations with immune checkpoint gene expression and tumor heterogeneity scores in LUAD. Functional studies demonstrate that *SEC61G* knockdown markedly inhibits the migration of A549 and H2030 LUAD cells. This inhibitory effect is accompanied by a significant downregulation of key regulators of tumor progression, including hypoxia-inducible factor-1 alpha (HIF-1α), lactate dehydrogenase A, and genes involved in the epithelial-mesenchymal transition pathway. In conclusion, our comprehensive analyses position SEC61G as a potential prognostic biomarker intricately linked to glycolytic metabolism, the EMT pathway, and the establishment of an immune-suppressive phenotype in LUAD. These findings underscore the potential of SEC61G as a therapeutic target and predictive marker for immunotherapeutic responses in LUAD patients.

## Introduction

Despite advances in diagnosis and treatment, recent research highlights lung adenocarcinoma (LUAD) as a leading cause of cancer-related deaths, especially in patients without driver gene mutations [ 1, 2]. As a result, understanding the molecular mechanisms and discovering new LUAD biomarkers are crucial.

SEC61 translocon subunit gamma (SEC61G) is a core subunit within the SEC61 complex located in the endoplasmic reticulum (ER) membrane and plays essential roles in ER protein translocation and cytoplasmic calcium (Ca ^2+^) homeostasis, especially in hypoxic microenvironments [ 3‒ 5]. In several cancers, including breast cancer, head and neck squamous cell carcinoma, glioblastoma, and kidney cancer, SEC61G is overexpressed and acts as a cancer promoter [ 6‒ 9]. Previous studies have also implicated SEC61G as an oncogene in lung cancer that contributes to ER stress, cell proliferation, apoptosis resistance, and EGFR activation [ 10‒ 13]. SEC61G is involved in ER protein translocation, and its overexpression can disrupt ER homeostasis, leading to ER stress and promoting adaptation to the stressful tumor microenvironment, aiding in tumor cell survival and chemoresistance [ 10, 13]. However, the potential associations between SEC61G expression and glucose metabolism, as well as epigenetic mechanisms in LUAD warrant further exploration.

SEC61G has been found to be associated with immune infiltration and the immune microenvironment in LUAD. It may modulate the immune response within the tumor by influencing the infiltration of immune cells such as T cells and macrophages [11]. A high level of SEC61G expression may affect the composition and function of immune cells within the tumor, potentially impacting the anti-tumor immune response. Interestingly, SEC61G is reportedly linked to metabolic reprogramming, such as glucose metabolism and glycolysis, in breast cancer cells [6]. This metabolic shift can provide cancer cells with a growth advantage and may impact the tumor microenvironment by altering nutrient availability and acidity.

In this study, we investigated the prognostic significance of SEC61G expression in pan-cancer patients using data from multiple independent datasets. Immunohistochemistry (IHC) was used to validate the differences in SEC61G expression between LUAD tissues and normal tissues. Enrichment analysis of co-expression genes was subsequently performed to investigate the underlying mechanisms of SEC61G’s involvement in LUAD pathogenesis. Correlations between SEC61G expression and immune infiltrating cells, as well as immune microenvironment modulators in LUAD, were examined. In addition, the effects of SEC61G expression on DNA variation and tumor heterogeneity in LUAD were evaluated. Furthermore, we analyzed the association between SEC61G expression and glycolysis in LUAD. Finally, experimental verification was performed to investigate the potential biological pathways affected by SEC61G in LUAD.

## Materials and Methods

### Ethics statement and clinical samples

The study was approved by the Ethics Committee of Fudan University, Shanghai Cancer Center, and was conducted in accordance with the Declaration of Helsinki. The study included 40 patients with lung adenocarcinoma (LUAD) who underwent surgery at Fudan University Shanghai Cancer Center. Formalin-fixed paraffin-embedded tumor tissues and adjacent normal tissues were collected from these patients, 20 of whom had undergone 18F-fluorodeoxyglucose positron emission tomography (18F-FDG-PET) before surgery. Furthermore, the potential correlation between SEC61G expression and glycolysis was evaluated by determining the maximum 18F-FDG standardized uptake value (SUV) in 20 patients with LUAD. We also investigated the correlation between the SEC61G immunohistochemistry (IHC) scores of surgically resected specimens and the SUV max values.

### Survival and expression analyses of SEC61G in pan-cancer and LUAD cohorts

The expression levels of SEC61G and its prognostic value were investigated in pan-cancer patients using The Cancer Genome Atlas (TCGA) database. Differences in SEC61G expression levels between LUAD tumor and normal tissues were also evaluated in the TCGA dataset ( *n*=594) and the GSE31210 dataset ( *n*=246). Correlations between SEC61G expression levels and the clinicopathological characteristics of LUAD patients were studied using the TCGA database.

### Enrichment analysis of the SEC61G gene co-expression network in LUAD

The statistical correlation between SEC61G expression and its co-expressed genes in the TCGA-LUAD cohort was calculated using Spearman’s correlation coefficient. Genes with a correlation coefficient (r)>0.35 or<‒0.35 and a *P* value<0.001 were selected. The results were visualized using volcano plots and heatmaps. Gene Ontology (GO) function and Kyoto Encyclopedia of Genes and Genomes (KEGG) pathway enrichment analyses of coexpressed genes were performed using the clusterProfiler and ggplot2 software packages in R software (4.2.0).

### Gene set enrichment analysis (GSEA)

GSEA was performed to investigate the biological processes associated with SEC61G in LUAD. An adjusted *P* value<0.001, a false discovery rate (FDR) *q* value<0.001, and a normalized enrichment score (NES)>1.5 were considered to indicate statistical significance. The “cluster Profiler” R package (3.8.0) was used for GSEA.

### Correlation between SEC61G and tumor immune infiltrating cells based on RNA-seq data in LUAD

Starting with preprocessed and normalized RNA-seq data from tumor samples from the TCGA dataset, we organized the data into a suitable matrix, with genes as rows and samples as columns. To assess the absolute proportion of tumor-infiltrated lymphocytes (TILs) in LUAD, we used the CIBERSORT deconvolution algorithm, input gene expression data, selected the relevant reference signature matrix and evaluated the proportion of immune cells using support vector regression. After execution, CIBERSORT provides estimates of immune cell proportions for each sample. The results were analyzed and visualized by conducting statistical tests and creating plots to compare immune cell infiltration between different sample groups, ultimately contributing to a better understanding of the immune composition of the TME. In addition, to evaluate the reliability of the deconvolution method, we used the “immuneeconv” R package, which provides an integrated *P* value from the six latest algorithms, TIMER, MCP-counter, CIBERSORT, EPIC and quanTIseq, for each sample. Single-sample gene-set enrichment analysis was conducted using the gene set variation analysis (GSVA) R package to quantify the relative abundance of 28 immune cell types [14].

### Relationship between genomic heterogeneity and SEC61G expression

We downloaded standardized generic data from the UCSC database TCGA Pan-Cancer (PANCAN, *n*=10535, G=60499), extracted the expression data of SEC61G in each sample, and further screened the sample sources from the Primary Blood-Derived Samples of Cancer-Peripheral Blood and Primary Tumors. We also downloaded the simple nucleotide variation data of all TCGA samples processed by “Mutect2” software from the GDC database. We used the tumor mutation burden (TMB) function of the R software package maftools (version 2.8.5) to calculate the TMB of each tumor, and we integrated the TMB and gene expression data of the samples. TMB, microsatellite instability (MSI), tumor purity, homologous recombination defect (HRD), and mutation allele heterogeneity (MATH) scores were evaluated to assess the association between SEC61G expression and DNA variation in LUAD. Stemness features associated with oncogenic dedifferentiation were evaluated and visualized using RNA-based stemness scores (RNA ss).

### Correlations of SEC61G expression with glycolysis- and m6A-related genes in LUAD

To further investigate the association between SEC61G expression and glycolysis, we conducted a correlation analysis between SEC61G expression and the expressions of glycolysis-related genes in two different datasets: GSE31210 and TCGA. The glycolysis-related genes we examined included *ENO1*, *G6PD*, *HK1*, *HK2*, *LDHA* , *LDHB*, *PDHB*, *PDK3*, *PDK4*, *PGK1*, *PKM*, *SLC2A1* , *SLC2A2*, and *SLC2A3*.

The m6A-related genes we examined included *ZC3H13*, *YTHDF3*, *HNRNPA2B1*, *IGF2BP1*, *IGF2BP3*, *YTHDC2*, *YTHDF1*, *FTO* , *HNRNPC*, *METTL14*, *METTL3*, *WTAP*, *RBM15*, *ALKBH5*, *IGF2BP2* , *RBMX*, *RBM15B*, *YTHDC1*, *VIRMA* and *YTHDF2*. We also analyzed the proportions of these genes in LUAD samples with high and low SEC61G expression. Kaplan-Meier curves were generated to assess the correlation between the expressions of these genes and the prognosis of LUAD patients.

### Immunohistochemistry

The tissue sections were incubated with a primary antibody specific for SEC61G (1:100, Df12136; Affinity, Chicago, USA) at 4°C overnight. Subsequently, the sections were incubated with an HRP-conjugated anti-rabbit IgG secondary antibody (1:2000, 656120; Thermo Fisher Scientific, Waltham, USA) for 50 min, followed by diaminobenzidine color development and counterstaining with hematoxylin after washing with water and phosphate-buffered saline. Image acquisition and analysis were performed, and the H-Score algorithm was utilized to determine the score, which was calculated as previously described [15]: percentage of cells with weak intensity×1+percentage of cells with moderate intensity×2+percentage of cells with strong intensity×3.

### Cell culture and transfection

Human non-small cell lung carcinoma H2030 and human lung adenocarcinoma A549 cell lines obtained from American Type Culture Collection (ATCC; Manassas, USA) were cultured in RPMI 1640 medium (Gibco, Carlsbad, USA) supplemented with 10% fetal bovine serum (FBS) and 1% penicillin/streptomycin (PS) (Thermo Fisher Scientific) under 5% CO _2_ at 37°C. To prevent mycoplasma infection, cells were treated with plasmin (Invitrogen, Carlsbad, USA). Small interfering RNAs (siRNAs) were designed using siRNA ( https://sg.idtdna.com/site/order/designtool/index/DSIRNA_CUSTOM) and BLOCK-iT™ RNAi Designer ( https://rnaidesigner.thermofisher.com/rnaiexpress/) ( Table 1) and transfected into H2030 and A549 cells using Lipofectamine 3000 (Invitrogen) following the manufacturer’s protocol. Knockdown efficiency was evaluated by qRT-PCR and western blot analysis 48 h post-transfection.

**Table:** **Table 1** Sequences of siRNAs used in gene knockdown experiment

Name	siRNA sequence (5′→3′)	Target
siSEC61G-NC	UUCUCCGAACGUGUCACGUTT (s) ACGUGACACGUUCGGAGAATT (as)	non-specific
siSEC61G-1	GUCGGCAGUUUGUAAAGGATT (s) UCCUUUACAAACUGCCGACTT (as)	SEC61G
siSEC61G-2	GGCAACAGCAAUAGGAUUU (s) AAAUCCUAUUGCUGUUGCC (as)	SEC61G

s: sense strand. as: antisense strand.

### Reverse transcription (RT) and quantitative real-time PCR analysis

The total RNA was extracted using the NucleoZOL (MACHEREY-NAGEL, Shanghai, China) according to the manufacturer’s instructions. RT was performed with 2 μg of total RNA using PrimeScript ^TM^ RT Master (TaKaRa, Dalian, China). The SYBR advantage qPCR premix (TaKaRa) was used for real-time quantitative PCR analysis. The sequences of primers used for RT-PCR were listed in Supplementary Table S1. Real-time fluorescence monitoring and a melting-curve analysis were performed with QuantStudio ^TM^7 Flex Real-Time PCR Software according to the manufacturer’s recommendations (QuantStudio ^TM^7 Flex Real-Time PCR System; Thermo Fisher Scientific). The samples were subjected to the establishment of three parallel wells each. The relative transcript abundance of the target gene was determined by normalizing the quantification obtained from serially diluted cDNA standard curves to that of *β-actin* within the same cDNA sample.

### Cell migration assay

A total of 3×10 ^4^ H2030 and A549 cells were suspended in 200 μL of RPMI 1640 without FBS and seeded into the top chamber of 24-well plate-sized transwell inserts (353097; BD Falcon, Franklin Lakes, USA) with an 8 μm pore size membrane. The lower chamber, which contained medium supplemented with 10% FBS, served as a chemoattractant. After incubation for 24 h, cells that did not migrate through the pores were manually removed with a cotton swab. Cells at the bottom of the membrane were stained with crystal violet and counted under a microscope (CKX31; Olympus, Tokyo, Japan). The cell numbers were calculated in eight random fields for each chamber, and the average value was calculated. Each experiment was conducted in triplicate.

### Scratch wound healing assay

Approximately 2×10 ^5^ H2030 and A549 cells were seeded onto 6-well plates and subjected to siRNA-mediated *SEC61G* knockdown for 48 h after cell attachment. Three fields of vision were randomly selected for each group, and images were taken under an optical microscope (ix71; Olympus) at 0 h and 36 h after wound induction.

### Protein extraction and western blot analysis

Proteins were extracted from harvested cells and separated by SDS-PAGE, and the proteins were subsequently transferred to polyvinylidene difluoride membranes. The membranes were blocked with 5% milk for 1 h at room temperature, followed by incubation with various primary antibodies overnight in 4°C, including rabbit polyclonal anti-SEC61G (1:1000, DF12136; Affinity), rabbit polyclonal anti-ZEB1 (1:1000, 21544-1-AP; Proteintech, Chicago, USA), rabbit monoclonal anti-HIF-1 alpha (1:1000, ab51608; Abcam, Cambridge, UK), rabbit monoclonal anti-LDHA (1:2000, 19987-1-AP; Proteintech), rabbit monoclonal anti-N-cadherin (1:1000, #13116; CST, Beverly, USA), rabbit monoclonal anti-E-cadherin (1:5000, 20874-1-AP; Proteintech), rabbit monoclonal anti-SNAIL (1:1000; CST), and mouse anti-actin (1:10,000, A5441; Sigma, St Louis, USA). After extensive wash, membranes were incubated with secondary antibodies (HRP-conjugated anti-rabbit IgG HRP/anti-mouse IgG) for 1 h at room temperature. Chemiluminescent detection was conducted using ECL reagent (ShareBio, Shanghai, China).

### Statistical analysis

Statistical analyses were performed using R software (3.8.0). Survival analysis was performed by Kaplan-Meier survival analysis, using the median value as the cut-off. A two-sided *P*<0.05 was considered to indicate statistical significance. The relative expression of SEC61G in the different groups was compared by the unpaired Student’s *t* test. The cut-off values for SEC61G expression in the high- and low-FDG-expression groups were determined based on the median value.

## Results

### SEC61G is a poor prognostic biomarker and is correlated with glucose metabolism in LUAD

**Figure 1:**
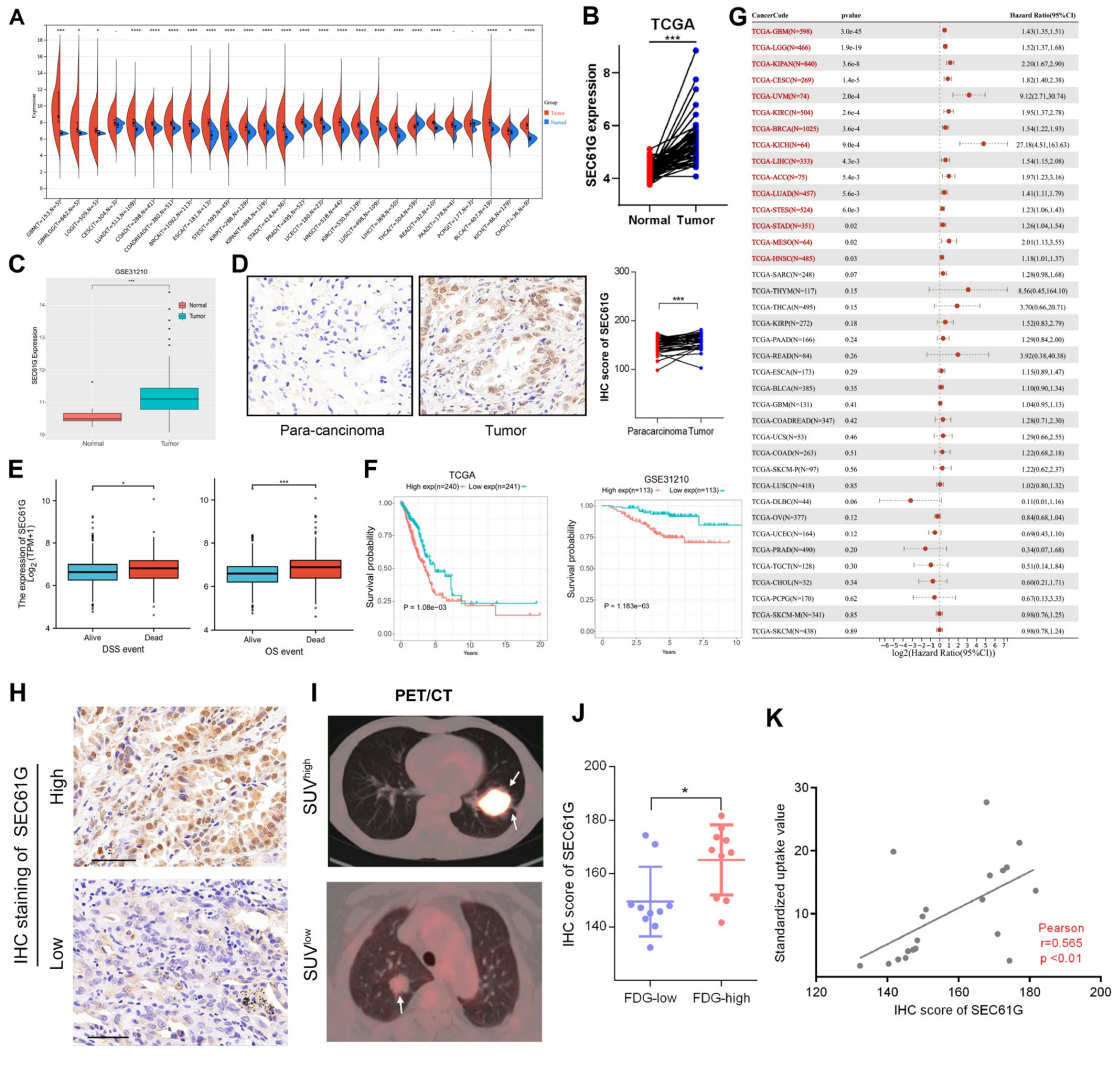
Figure 1 SEC61G tends to predict poor OS and is associated with glucose metabolism in lung adenocarcinoma (LUAD) patients (A) SEC61G mRNA expression levels across cancers. (B,C) Differences in the expression of SEC61G between LUAD tissues and matched normal tissues in the TCGA database (B) and the GSE31210 dataset (C). (D) Immunohistochemistry results showing the expression of SEC61G in LUAD tissues and para-carcinoma tissues. Magnification, 200×. (E) DSS and OS events based on the expression of SEC61G. (F) The survival curves of patients stratified according to SEC61G expression in the TCGA and GSE31210 datasets. (G) OS analyses of patients stratified by the SEC61G mRNA expression level across cancers based on the TCGA datasets. (H,I) Representative SEC61G immunohistochemical images (H) and PET/CT images (I) with high FDG uptake and low FDG uptake. Scale bar: 50 μm. (J) Statistical analysis of SEC61G expression in LUAD patients with high FDG uptake and patients with low FDG uptake. (K) Correlation between FDG uptake and SEC61G expression in 40 LUAD patients. * P<0.05, ***P<0.001, ****P <0.0001.

This study aimed to investigate the expression pattern and prognostic significance of SEC61G in various cancers using data from the TCGA cohort. We analyzed RNA sequencing data to evaluate the expression of SEC61G in different types of tumor tissues and revealed that SEC61G is overexpressed in many types of tumor tissues as well as in LUAD ( Figure 1A,B). Furthermore, the expression levels of SEC61G in LUAD were also confirmed by the GSE31210 dataset and the IHC results of our cohort ( Figure 1C,D). In addition, among OS events ( *P*<0.01) and DSS events ( *P*<0.05), SEC61G expression is significantly greater in the nonsurviving group than in the surviving group ( Figure 1E). Moreover, SEC61G tends to be a poor prognostic predictor in LUAD and many other kinds of cancers ( Figure 1F,G). Multivariate Cox regression analysis indicated that SEC61G is an independent poor prognostic factor in LUAD patients [HR=1.338 (1.050‒1.706), *P*=0.018] ( Supplementary Tables S2and 3). Furthermore, we observed that SEC61G is more highly expressed in lesions with high FDG uptake ( Figure 1H,I). The correlation between SEC61G expression and FDG uptake was verified by the IHC scores of clinical samples ( Figure 1J,K). Overall, these findings provide evidence that SEC61G is overexpressed in various cancers and is a poor prognostic factor in several cancers, including LUAD. These findings suggest that SEC61G could be a potential therapeutic target in LUAD and may be associated with glucose metabolism.

### Correlations of SEC61G expression with glycolysis in LUAD

**Figure 2:**
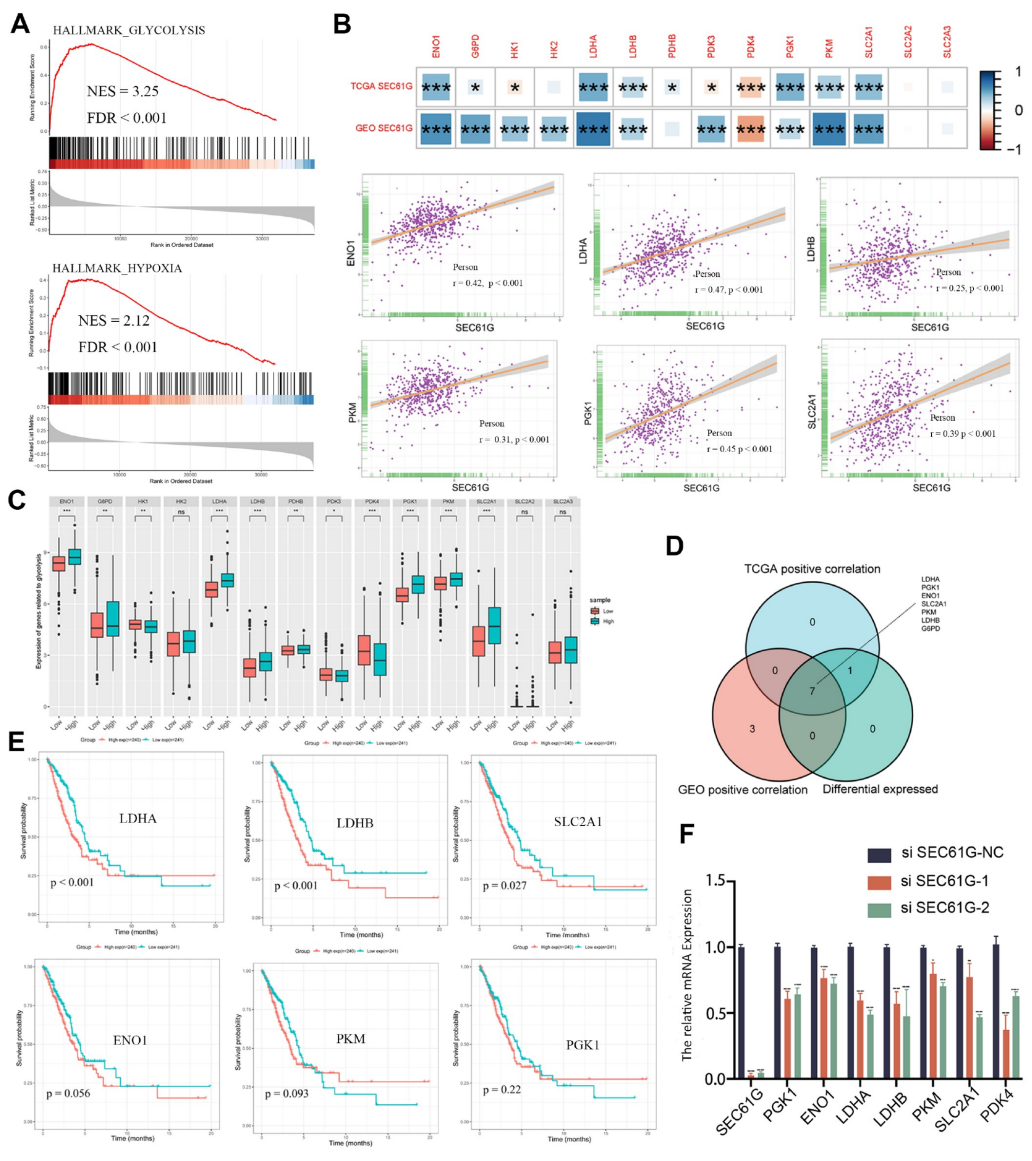
Figure 2 Correlations of SEC61G expression with glycolysis-related genes in LUAD (A) Glycolysis and hypoxia pathways were enriched in the SEC61G-high group based on the TCGA database. (B) Correlation analysis of SEC61G and glycolysis-related gene expression based on the GSE31210 and TCGA LUAD datasets. Scatter plots showing the correlations between the top 6 glycolysis-related genes. (C) The differential expressions of glycolysis-related genes between the high and low SEC61G expression groups in LUAD tumor samples. (D) Venn diagram showing both the expression correlation and differential expression of genes, including LDHA, LDHB , SLC2A1, ENO1, PKM, PGK1 and G6PD. (E) Kaplan-Meier curves of ENO1, HK2, LDHA, LDHB, PGK1 and SLC2A1 expression. (F) The expressions of glycolysis-related genes following SEC61G knockdown was measured by qRT-PCR in A549 cells. *P<0.05, **P<0.01, ***P<0.001, ****P<0.0001. ns, not significant.

To explore the potential mechanisms of SEC61G in LUAD, we conducted GSEA on the DEGs between the SEC61G high- and low-expression groups in LUAD. The results showed that glycolysis (NSE=3.25, *P*<0.001) and hypoxia (NSE=2.12, *P*<0.001) are enriched in the SEC61G-high group ( Figure 2A). Then, we investigated the correlation between SEC61G expression and 14 glycolysis-related genes in the GSE31210 and TCGA LUAD datasets. Our results indicate that SEC61G expression is positively correlated with the expressions of ENO1, G6PD, LDHA, LDHB, PGK1, and SLC2A1 but negatively correlated with PDK4 expression in both datasets ( Figure 2B). A scatter plot of SEC61G and glycolysis-related genes revealed a positive correlation, especially with *ENO1*, *LDHA*, *LDHB*, *PKM* , *PGK1*, and *SLC2A1* ( Figure 2B). Moreover, the expressions of ENO1, G6PD, HK2, LDHA, LDHB, PDHB, PGK1, PKM, and SLC2A1 are elevated in the high SEC61G expression group ( *P*<0.05; Figure 2C). The Venn diagram illustrates the genes that are correlated with both datasets and the genes differentially expressed according to SEC61G, including *ENO1*, *G6PD*, *LDHA*, *LDHB* , *PKM*, *PGK1*, and *SLC2A1* ( Figure 2D). Furthermore, our Kaplan-Meier curves showed that high expressions of LDHA, LDHB, and SLC2A1 are strongly associated with poor prognosis in patients with LUAD ( Figure 2E; *P*<0.05). Moreover, we evaluated the mRNA expression levels of glycolysis-related genes, including *ENO1*, *LDHA*, *LDHB*, *PGK1* , *PKM*, *SLC2A1*, and *PDK4*, after the knockdown of *SEC61G*. The results showed that the mRNA expression levels of LDHA, LDHB, and SLC2A1 in the A549 cell line decreased most significantly determined by RT-qPCR ( Figure 2F). Therefore, our findings suggest that SEC61G may be involved in glycolysis in LUAD, particularly through the regulation of LDHA, LDHB, and SLC2A1.

### Knockdown of *SEC61G* suppresses lung cancer cell migration and promotes EMT *in vitro*

**Figure 3:**
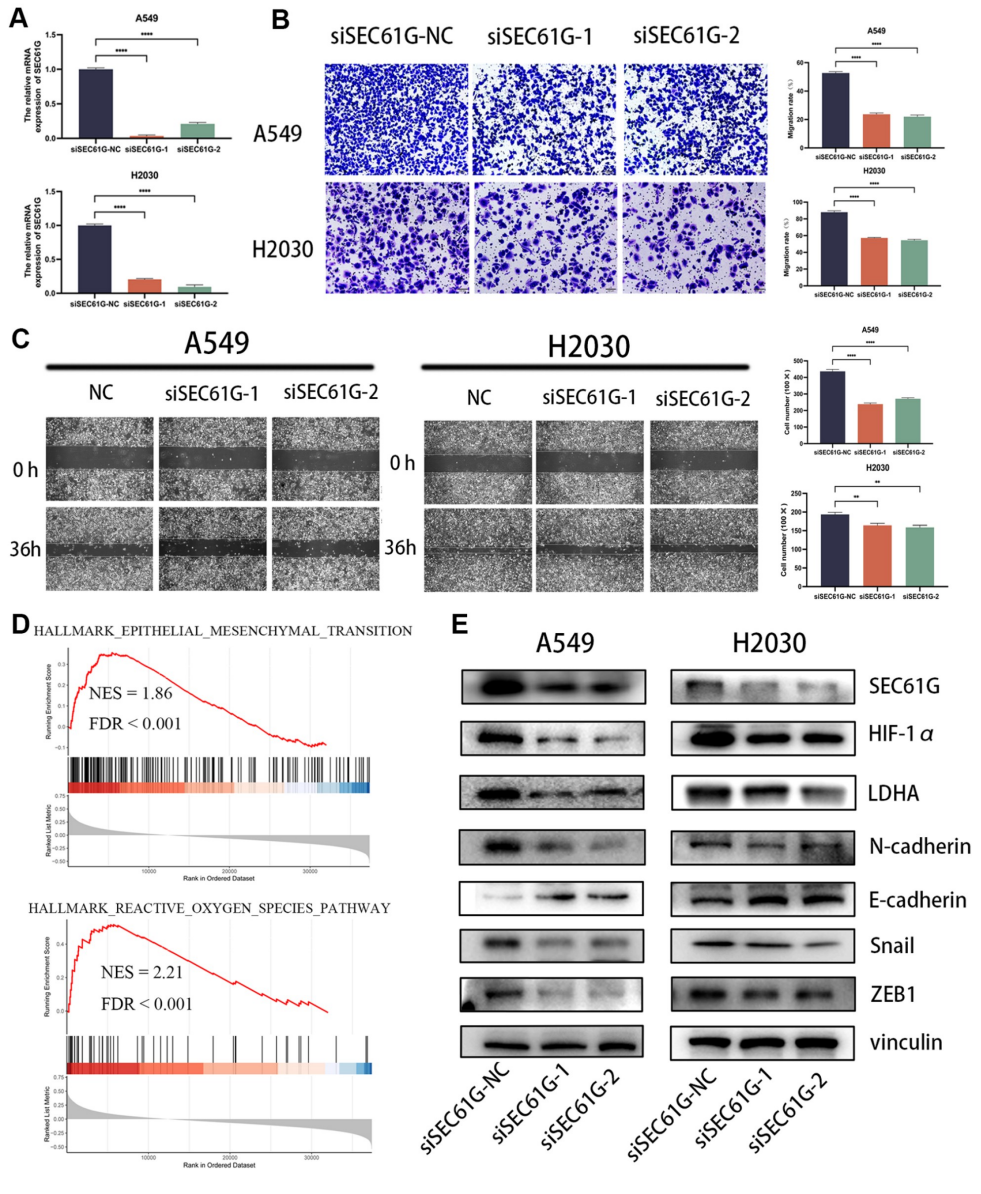
Figure 3 Knockdown of *SEC61G* suppresses lung cancer cell migration and promotes EMT *in vitro* (A) The effect of SEC61G knockdown on A549 cells and H2030 cells was measured by qRT-PCR. (B) Migration assays showed that knockdown of SEC61G significantly decreased the number of migrated cells. Magnification, 200×. (C) Wound healing assays showed that knockdown of SEC61G significantly decreased the number of migrated cells within 36 h. (D) The epithelial mesenchymal transition and reactive oxygen species pathways were enriched in the SEC61G-high group according to the TCGA database. (E) Knockdown of SEC61G decreased the protein expressions of HIF-1α, LDHA, and EMT-related genes. **P<0.01, ****P <0.0001.

The present study aimed to investigate the role of SEC61G in regulating lung cancer cell migration *in vitro*. The effect of *SEC61G* knockdown on A549 and H2030 lung cancer cell lines was assessed using quantitative RT-qPCR ( Figure 3A). Migration assays, including scratch wound healing and transwell assays, were conducted to assess the impact of *SEC61G* knockdown on cell migration. In A549 and H2030 cells, the relative migration ratio significantly decreased by 59.62% ( *P*=0.0092) and 63.73% ( *P*=0.0019), respectively ( Figure 3B). Additionally, the scratch wound healing assay revealed decreases in the relative migration ratios of 33.32% ( *P*=0.0026) and 38.65% ( *P*<0.001) in the A549 and H2030 cells, respectively ( Figure 3C). Moreover, enrichment analysis of the TCGA database indicated that high SEC61G expression is associated with EMT (NSE=1.85, *P*<0.001) and reactive oxygen species (ROS) (NSE=2.21, *P*<0.001) ( Figure 3D).

To determine the underlying molecular mechanisms involving SEC61G in hypoxia and the EMT process, we conducted western blot analysis to assess changes in the levels of proteins downstream of SEC61G. The results demonstrated a significant reduction in LDHA, HIF-1α, and EMT-related proteins ( Figure 3E). These findings suggest that SEC61G may enhance the migratory ability of lung cancer cells by modulating the expressions of glycolysis-related and EMT-related genes. Further research is required to elucidate the specific molecular pathways implicated in this process.

### Differential expression and functional enrichment of SEC61G and its role in the tumor immune microenvironment in LUAD

**Figure 4:**
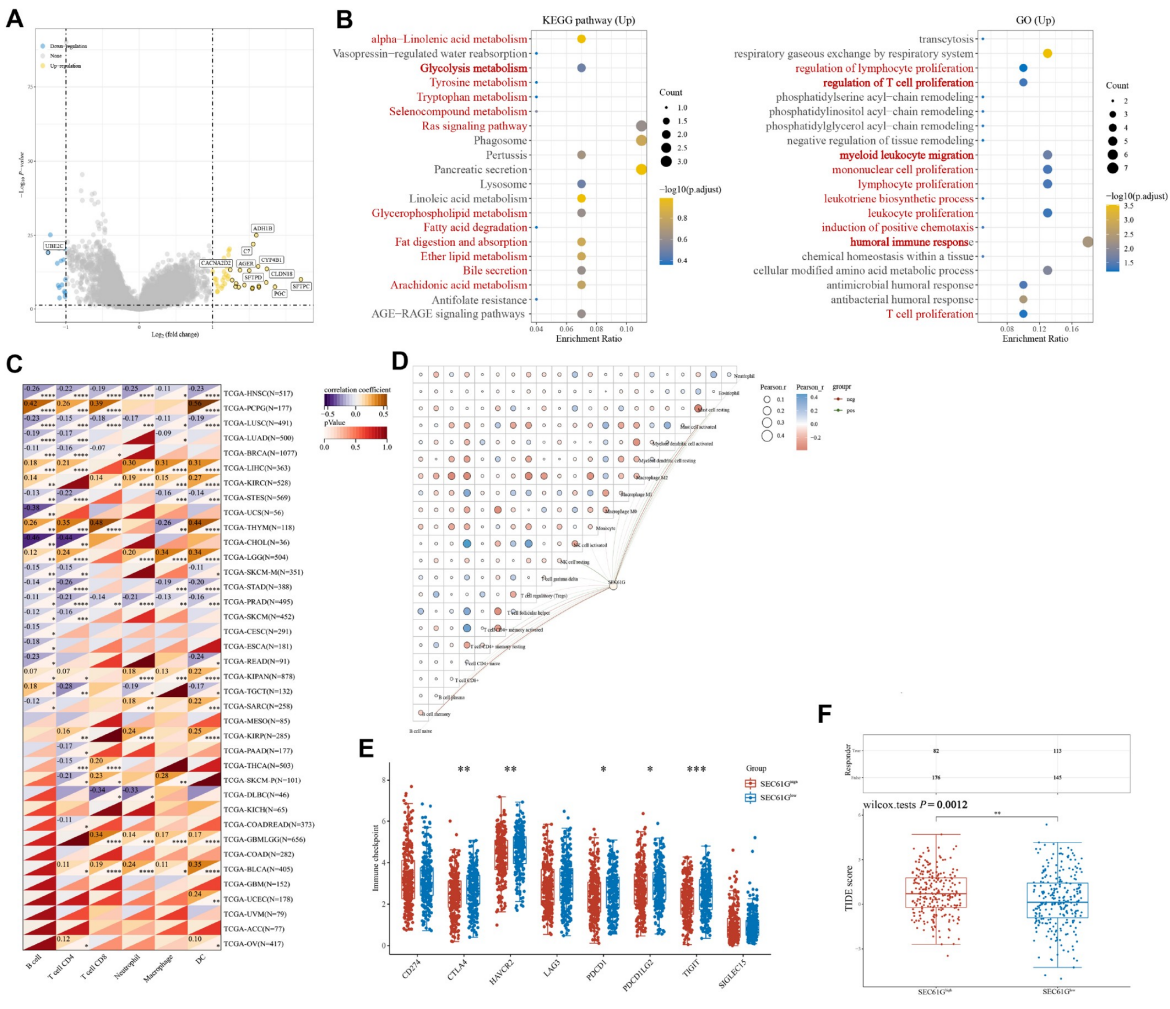
Figure 4 Correlation between SEC61G and tumor immune infiltrating cells (A) Volcano plot showing coexpressed genes associated with SEC61G expression. Correlations between the expression of SEC61G and immune infiltrating cells in LUAD according to the TIMER database. (B) Enrichment analysis of GO terms and KEGG terms for genes coexpressed with SEC61G. The SEC61G CNV affected the infiltration levels of B cells, CD4+ T cells, CD8+ T cells, macrophages, and dendritic cells in LUAD patients. (C) The relationship between SEC61G expression and immune cell infiltration in the tumor microenvironment across cancers. (D) Pearson’s correlation between SEC61G expression and the abundance of immune cells in the LUAD tumor microenvironment. (E) Correlations between immune checkpoint molecule expression and SEC61G expression. (F) The relationship between SEC61G expression and the TIDE score in LUAD. *P<0.05, **P<0.01, ***P<0.001.

In this study, we aimed to investigate the detailed roles of SEC61G in LUAD progression. We analyzed gene expression patterns in LUAD and identified significantly upregulated genes, including *ADH1B*, *C7* , *CYP4B1*, *CLDN18*, *PGC*, and *SFTPC*, among others, associated with SEC61G expression ( Figure 4A). Additionally, KEGG enrichment analysis indicated increased metabolic activities, including glycolysis metabolism, the Ras signaling pathway, and fatty acid metabolism ( Figure 4B), while GO enrichment analysis highlighted enhanced T-cell proliferation, myeloid leukocyte migration, and humoral immune responses ( Figure 4B). Using the CIBERSORT algorithm, we assessed the relationship between SEC61G expression and T-cell infiltration in the tumor microenvironment of LUAD patients ( Figure 4C). Pearson’s correlation analysis revealed a strong association between SEC61G expression and the abundance of immune cells in the LUAD tumor microenvironment ( Figure 4D). To explore the potential of SEC61G expression as a predictor of LUAD patient response to immune checkpoint inhibitor (ICI) treatment, we analyzed the expressions of immune checkpoint molecules and found that CTLA4, PD-1, and TIGIT were significantly downregulated in patients with high SEC61G expression ( Figure 4E). Furthermore, patients with higher SEC61G expression had significantly greater TIDE scores ( *P*=0.0012), indicating greater intratumoral heterogeneity ( Figure 4F). These findings provide insights into the potential role of SEC61G in LUAD progression and its implications for immunotherapy response.

### SEC61G expression predicts the differential DNA variation landscape of LUAD

**Figure 5:**
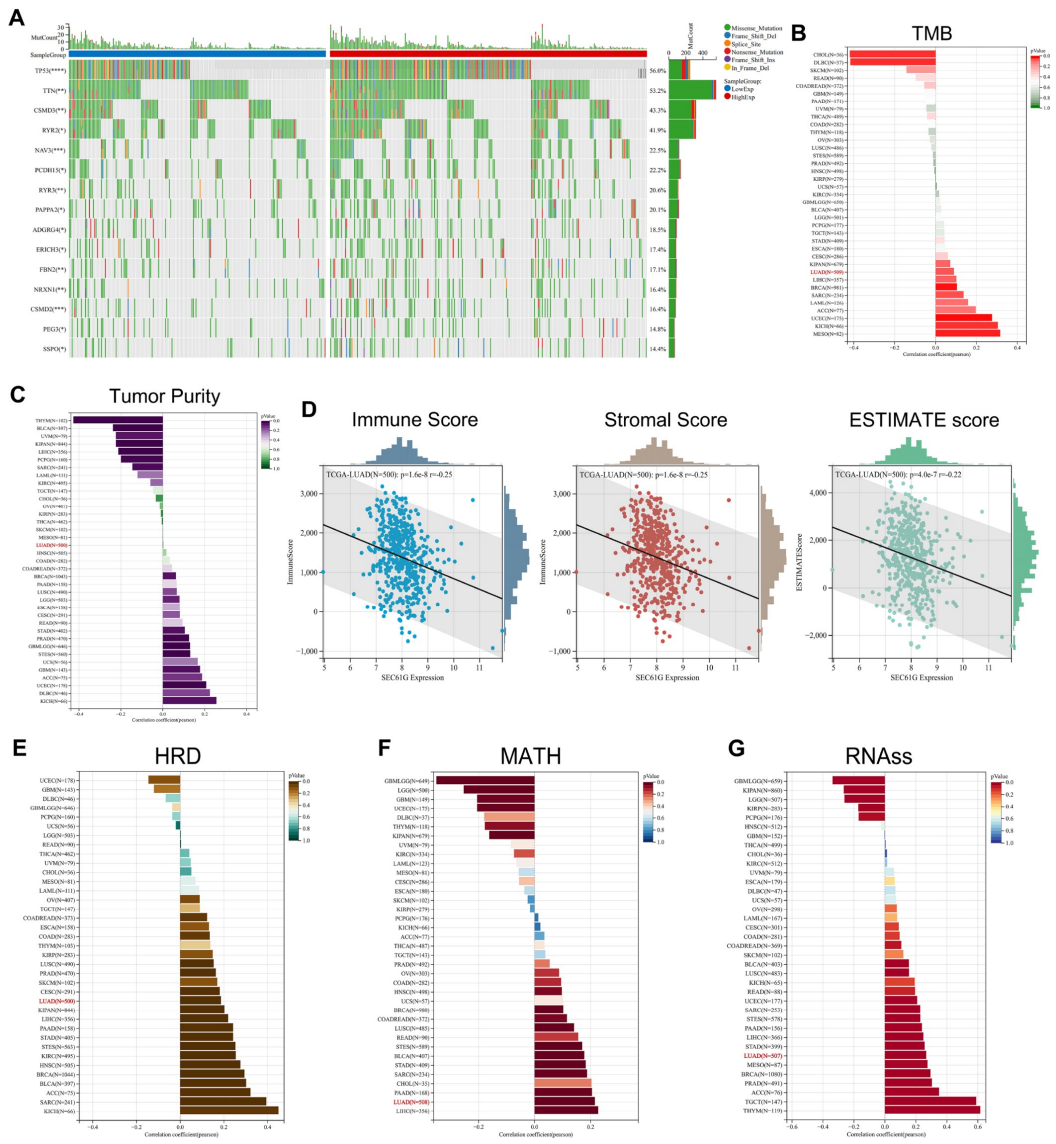
Figure 5 SEC61G expression predicts the differential DNA variation landscape of LUAD (A) The DNA variation landscape in relation to SEC61G expression in LUAD. (B) The relationship between SEC61G expression and TMB across various cancer types. (C) The correlations between SEC61G expression and tumor purity. (D) Correlations between SEC61G expression and immune score, stromal score, and ESTIMATE score. (E–G) Correlations between SEC61G expression and homologous recombination deficiency (HRD) (E), mutant-allele tumor heterogeneity (MATH) score (F), and ribonucleic acid structure similarity (RNA ss) (G).

In this study, our primary objective was to investigate the DNA variation landscape in relation to SEC61G expression in LUAD. As illustrated in Figure 5A, we stratified LUAD patients into groups with either high or low SEC61G expression. Subsequently, we observed significant differential genomic variations in genes such as *TP53* , *TTN*, *CSMD3*, *RYR2*, *NAV3*, *PCDH15*, *RYR3*, *PAPPA2* , *ADGRG4*, *ERICH3*, *FBN2*, *NRXN1*, *CSMD2*, and others. These variations warrant further investigation to determine their potential roles in LUAD pathogenesis.

Spearman’s correlation analysis was used to explore the relationship between SEC61G expression and TMB across various cancer types. Intriguingly, we identified a significant positive association between SEC61G expression and TMB, specifically in LUAD ( Figure 5B). This finding suggests a unique genomic landscape in LUAD that may be influenced by SEC61G. Furthermore, we assessed the impact of SEC61G expression on the tumor microenvironment and genetic instability. To further explore these associations within the context of LUAD, we utilized the ESTIMATE algorithm. Our analysis revealed a negative correlation between SEC61G expression and immune score, stromal score, as well as ESTIMATE score ( Figure 5C,D). Additionally, SEC61G expression exhibits a positive correlation with increased homologous recombination deficiency (HRD), mutant-allele tumor heterogeneity (MATH) score, and ribonucleic acid structure similarity (RNA ss) in LUAD ( Figure 5E‒G). These findings shed light on the intricate relationship between SEC61G expression and various genomic and immune-related factors in LUAD. Further investigations are warranted to decipher the clinical and biological implications of these associations and their potential role in LUAD progression and prognosis.

### Correlations of SEC61G expression with m6A modification in LUAD

N6-methyladenosine (m6A) modification has been reported to play a crucial role in the development of lung adenocarcinoma (LUAD) [ 16, 17]. In this study, we aimed to investigate the potential relationship between SEC61G expression and the expression of 20 m6A-related genes in LUAD. We conducted a comprehensive analysis using two distinct datasets, as mentioned above, and applied rigorous methodologies for gene expression analysis and statistical testing.

**Figure 6:**
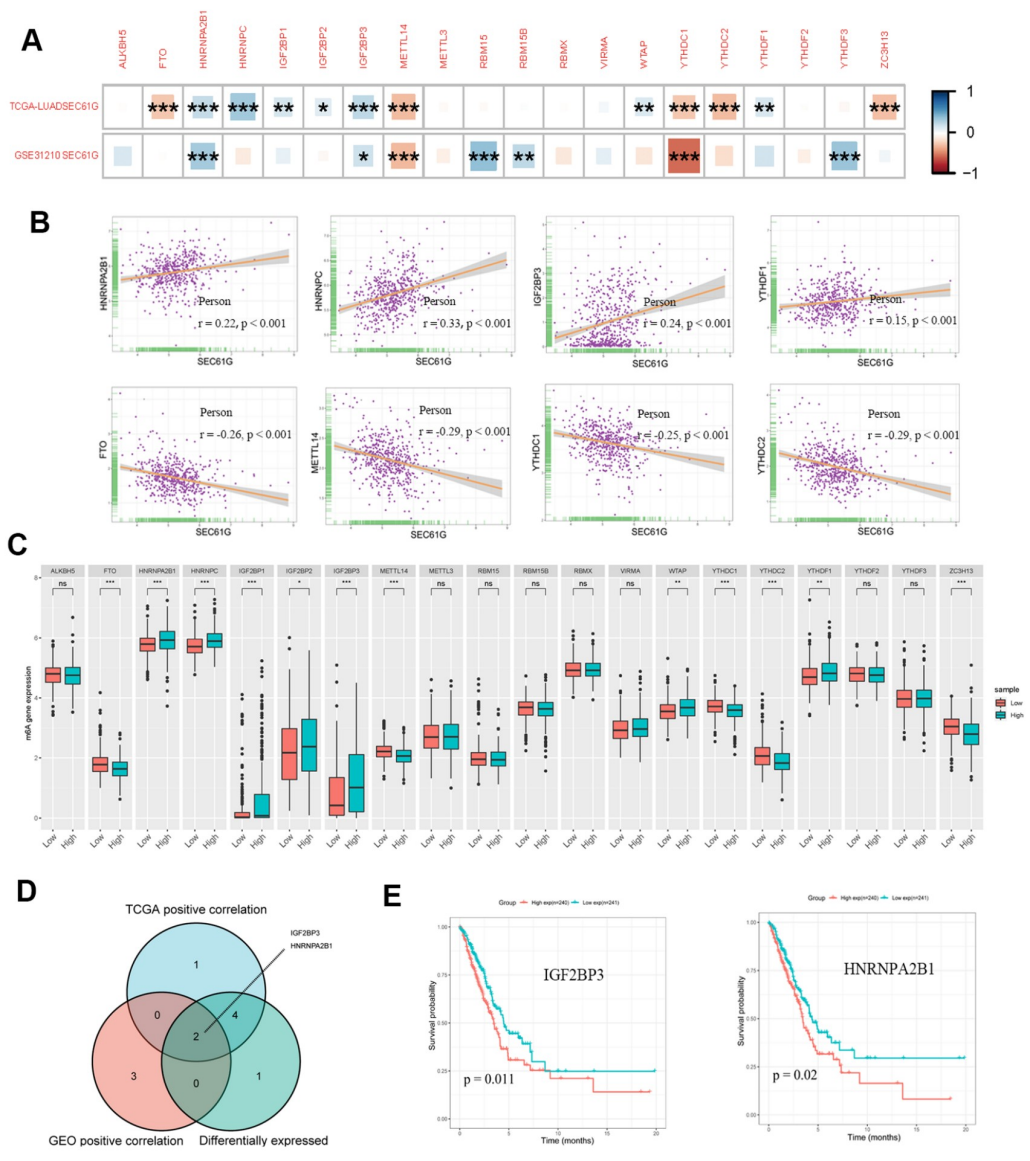
Figure 6 Correlations of SEC61G expression with m6A-related genes in lung adenocarcinoma (LUAD) (A) The GSE31210 and TCGA LUAD datasets were used to analyze the correlation between SEC61G and m6A-related gene expression in LUAD. (B) Scatter plots showing the correlation between SEC61G and m6A-related gene expression. (C) The differential expressions of m6A-related genes in the high and low SEC61G expression groups in LUAD. (D) Venn diagram showing both the expression correlation and differential expression of genes, including IGF2BP3 and HNRNPA2B1. (E) Kaplan-Meier curves of IGF2BP3 and HNRNPA2B1 expressions. *P<0.05, **P<0.01, ***P<0.001. ns, not significant.

Our results revealed a significant positive correlation between SEC61G expression and the expression of HNRNPA2B1 and IGF2BP3. Conversely, SEC61G expression is negatively correlated with METTL14 and YTHDC1 expression in both datasets ( Figure 6A; *P*<0.05). To visualize these relationships, a scatter plot illustrating the correlation between SEC61G expression and m6A-related gene expression in the TCGA database was generated ( Figure 6B). Furthermore, we stratified the TCGA LUAD samples into high- and low-expression groups based on the SEC61G level. Our analysis demonstrated significantly greater expression of HNRNPA2B1, HNRNPC, IGF2BP1, IGF2BP2, IGF2BP3, WTAP, and YTHDF1 in the high-expression group of SEC61G than in the low-expression group ( Figure 6C; *P*<0.05). Notably, the Venn diagram ( Figure 6D) revealed overlapping differentially expressed genes between SEC61G and m6A-related genes, including *HNRNPA2B1* and *IGF2BP3* .

To assess the clinical relevance of these findings, Kaplan-Meier curves were generated, and the results demonstrated that high expression of HNRNPA2B1 and IGF2BP3 is associated with poor prognosis in patients with LUAD ( Figure 6E; *P*<0.05). Overall, *HNRNPA2B1* and *IGF2BP3* are potential interacting genes related to the involvement of SEC61G in the progression of lung cancer, but further experimental validation is needed. The interaction between SEC61G and these m6A-related genes may have significant implications for understanding the molecular mechanisms underlying LUAD development and progression. Further research is needed to elucidate the precise mechanisms by which SEC61G influences m6A modification in this context.

## Discussion

*SEC61G* encodes the γ subunit of the SEC61 complex, which functions as a channel across the endoplasmic reticulum (ER) membrane, playing a pivotal role in nucleocytoplasmic signal transport within the ER and the ER-Golgi intermediate compartment [ 3 , 18, 19]. Additionally, the Sec61 translocon has been identified as a regulator of Ca ^2+^ leakage from the ER, potentially impacting mitochondrial ROS production and ATP synthesis [ 19‒ 21]. However, the precise roles of SEC61G in immune function and metabolism in LUAD remain to be elucidated. Consistent with prior studies, our analysis revealed increased expression and poor prognosis in many cancers, including LUAD [ 7, 11, 22]. Our results were corroborated through cross-referencing with various online databases and validated by immunohistochemical staining in our own cohort. Therefore, SEC61G clearly holds promise as a potential diagnostic and prognostic marker for LUAD, corroborating earlier research [ 11, 13].

To further explore the biological implications of SEC61G, we examined its co-expressed genes. KEGG analysis highlighted enrichment in pathways related to glycolysis within the SEC61G-up subgroup. Gene Ontology (GO) enrichment analysis suggested involvement in immune cell proliferation and migration. Additionally, GSEA revealed upregulated genes associated with pathways involved in glycolysis, hypoxia, EMT and ROS production. The induction of the EMT pathway, a process intricately linked to the aggressiveness and dissemination of tumors, can be precipitated by HIF-1α under hypoxic conditions, indicating a prognostically adverse association with oncological outcomes [ 23, 24]. Since the endoplasmic reticulum and mitochondria are the main organelles responsible for the production of ROS, we speculated that SEC61G may influence carbohydrate metabolism and the EMT process through complex interactions between the ER and mitochondria. While excessive ROS can induce ferroptosis in tumor cells, playing a role in cancer suppression, ROS can also promote tumor progression and metastasis by stabilizing HIF1α [25]. This potential mechanism could be attributed to the leakage of Ca ^2+^ from the ER to the mitochondria, a phenomenon known to induce ROS production and facilitate cell migration in lung cancer cells [26]. These findings shed light on the multifaceted role of SEC61G in LUAD, suggesting its involvement in critical cellular processes beyond its established functions.

The promotion of glycolysis is strongly associated with cancer development and poor prognosis. Previous research has revealed that SEC61G promotes tumorigenesis through the glycolysis and EMT pathways in breast cancer patients [ 6, 27]. In our study, bioinformatics analysis revealed a significant positive correlation between *SEC61G* expression and the expressions of genes such as *LDHA* , *PKM*, *ENO1*, *SLC2A1*, *G6PD* , *HK1*, *PDK3*, *LDHB*, and *HK2*. Kaplan-Meier curve analyses demonstrated that LUAD patients with elevated LDHA, LDHB, and SLC2A1 expression tended to have poorer prognoses. LDHA and LDHB were thus identified as poor prognostic factors in LUAD patients, linking glycolysis and oxidative phosphorylation and the dynamic interplay between these two metabolic states [ 28‒ 31]. Furthermore, SLC2A1, also known as GLUT1, is associated with an unfavorable prognosis in LUAD patients and contributes to premetastatic niche formation, impacting innate immune cells [ 32, 33].

The immune infiltration of tumor cells is closely related to lymph node metastasis and LUAD prognosis [ 34, 35]. Our findings indicated that SEC61G is positively correlated with lymph node metastasis ( Supplementary Table S3) but negatively correlated with B cells, CD4+ T cells, macrophages, and dendritic cells. In summary, SEC61G may trigger an antitumor immune response by inhibiting the antigen-presenting cells mentioned above. This effect might be attributed to the reduced expressions of MHC class I molecules in the high SEC61G group, which aligns with the known role of SEC61G in stabilizing immune checkpoint ligands (ICLs), including PD-L1, PVR, and PD-L2, through glycosylation in EGFR-amplified glioblastoma multiforme (GBM) [ 36‒ 38]. These findings underscore the significant role of SEC61G in shaping a suppressed immune microenvironment in LUAD.

m6A is the most prevalent internal modification of mRNAs and has been implicated in the pathological processes associated with carcinogenesis, including uncontrolled cell proliferation, migration, invasion, and EMT [ 39‒ 41]. However, there is limited research on the relationship between SEC61G and m6A in solid tumors. In our study, we observed that high expression levels of HNRNPA2B1 and IGF2BP3 may serve as indicators of a poor prognosis in LUAD patients. This observation aligns with previous reports, as HNRNPA2B1 has been associated with poor prognosis in lung cancer through EMT and its role in shaping a premetastatic microenvironment [ 42‒ 44]. Additionally, IGF2BP3 has been linked to alternative splicing of multiple genes, particularly those clustered in response to hypoxia, and it is correlated with nodal metastasis and brain metastasis [ 45‒ 47].

Furthermore, our *in vitro* experiments confirmed that the knockdown of *SEC61G* could reduce the migration and invasion of lung cancer cells. Based on these results, we hypothesized that SEC61G might interact with LDHA and EMT-related signaling pathways. Consequently, we conducted a series of *in vitro* experiments in two cell lines to test this hypothesis. The results demonstrated that the knockdown of *SEC61G* led to decreased expressions of *HIF-1α*, *LDHA*, and EMT-related genes. This finding suggested that SEC61G may promote cancer progression and metastasis through HIF-1α, LDHA, and EMT-related pathways. This finding may be attributed to the increased expression levels of mitochondrial ROS and HIF-1α subsequent to Ca ^2+^ leakage, which subsequently promotes the expressions of LDHA and EMT-related molecules [ 48‒ 50].

In conclusion, our comprehensive analyses position SEC61G as a potential prognostic biomarker intricately linked to glycolytic metabolism, the EMT pathway, and the establishment of an immune-suppressive phenotype in LUAD. These findings not only enhance our understanding of LUAD pathogenesis but also underscore the potential of SEC61G as a therapeutic target and predictive marker for immunotherapeutic responses in LUAD patients.

## Supplementary Data

Supplementary data is available at *Acta Biochimica et Biphysica Sinica* online.
